# Co‐infection by *Cryptococcus neoformans* fungaemia and non‐tuberculous mycobacteria with *Pneumocystis jiroveci* pneumonia in a newly diagnosed HIV‐infected patient

**DOI:** 10.1002/ccr3.4191

**Published:** 2021-05-05

**Authors:** Eihab A. Subahi, Mohamad S. Aljafar, Haidar H. Barjas, Mohamed Abdelrazek, Fatima A. Rasoul

**Affiliations:** ^1^ Department of Internal Medicine Hamad Medical Corporation Doha Qatar; ^2^ Department of Radiology Hamad Medical Corporation Doha Qatar

**Keywords:** Cryptococcosis, human immunodeficiency virus, Mycobacterium, Pneumocystis pneumonia

## Abstract

Opportunistic infections are common in human immunodeficiency virus (HIV)‐infected patients. Co‐infections with *Cryptococcus neoformans* and *Mycobacterium* species together with *Pneumocystis jiroveci* pneumonia (PCP) are rare and typically occur in immunocompromised individuals, particularly acquired immunodeficiency syndrome patients.

## Graphical abstract

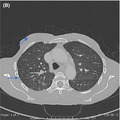

## 1 INTRODUCTION

Opportunistic infections caused by bacteria and fungi are common in human immunodeficiency virus (HIV)‐infected patients. Co‐infections with *Cryptococcus neoformans* and *Mycobacterium* species together with *Pneumocystis jiroveci* pneumonia (PCP) are rare and typically occur in immunocompromised individuals, particularly acquired immunodeficiency syndrome patients. Herein, we report the case of a 51‐year‐old man who presented with a 20‐day history of productive cough, fever, and progressive shortness of breath associated with significant weight loss; he was found to have HIV infection. Further investigations revealed *C*. *neoformans* fungaemia together with mycobacterial infection and PCP, in addition to other infections.

The human immunodeficiency virus (HIV) and acquired immunodeficiency syndrome (AIDS) pandemic have become a well‐known global health problem, especially in the last decade. Due to the effectiveness of highly active antiretroviral therapy (HAART), the incidence of new HIV infections has declined, and the number of AIDS‐related deaths in adults and children worldwide has plateaued.^1^

Many of the clinical features of HIV/AIDS can be ascribed to the profound immune deficiency that develops in infected patients. The destruction of the immune system by the virus results in opportunistic infections, as well as an increased risk of autoimmune disease and malignancy. HIV‐related complications are rarely encountered in patients with preserved immunity (CD4 T‐cell counts greater than 500 cells/mm^3^). The risk of developing opportunistic infections and malignancies typical of AIDS increases progressively as the CD4 count falls below 200 cells/mm^3^.^2^

Meanwhile, the respiratory system is one of the most frequently affected organ systems in HIV‐infected patients, and opportunistic pulmonary infections remain a major threat.^3^ The pathogens may be bacteria, mycobacteria, fungi, or viruses.

## 2 CASE PRESENTATION

A 51‐year‐old man with no significant past medical history presented with a 20‐day history of cough productive of yellowish sputum which was sometimes blood streaked. He also reported nocturnal fever, progressive shortness of breath accompanied with generalized fatigue, and significant unintentional weight loss of approximately 15 kg, with loss of appetite. He had no history of any other symptom and no history of intravenous drug use or tattoos. He was a construction worker, married, but admitted to having a few extra‐marital sexual relationships.

On examination, he was febrile with a temperature of 38.9°C; other vital signs were within normal limits. The patient was cachectic and pale. The examination of the neck was unremarkable. Chest examinations showed reduced air entry in the right lower lung zone with coarse crackles and dull percussion notes. The rest of the physical examination was unremarkable.

His laboratory findings on admission showed bicytopenia (anemia and thrombocytopenia), with an Hb level of 8.5 mg/dL and platelet count of 127 × 10^3^/µL. Later on, he developed pancytopenia with white blood cells count reaching 1.9 × 10^3^/µL (absolute neutrophil count was 1.5 × 10^3^/µL, and lymphocyte count was 0.3 × 10^3^/µL). Liver function test and renal function test were normal. A peripheral blood smear showed normocytic normochromic anemia with thrombocytopenia and lymphopenia with few reactive lymphocytes. Blood testing showed positive results for HIV, and accordingly, other tests were requested. Sputum smear for acid‐fast bacilli and PCR showed negative results for tuberculosis (TB). Meanwhile, two weeks later, his sputum culture was positive for nontuberculous mycobacteria (NTM), and at the same time, his Quantiferon test showed indeterminate results. Bronchoscopy with bronchoalveolar lavage (BAL) was performed, and 2 weeks later, BAL culture was positive for NTM, *P*. *jiroveci,* and *Candida albicans*. Both cytomegalovirus (CMV) and Epstein‐Barr virus (EBV) PCR tests using BAL aspirates showed positive results. Furthermore, blood culture showed the growth of *C*. *neoformans*. Rapid plasma reagin screening for syphilis showed positive results, and a confirmatory test for *Treponema pallidum* antibodies also showed positive results. Urine tests for *Chlamydia trachomatis* and *Neisseria gonorrhoeae* DNA showed negative results.

**FIGURE 1:**
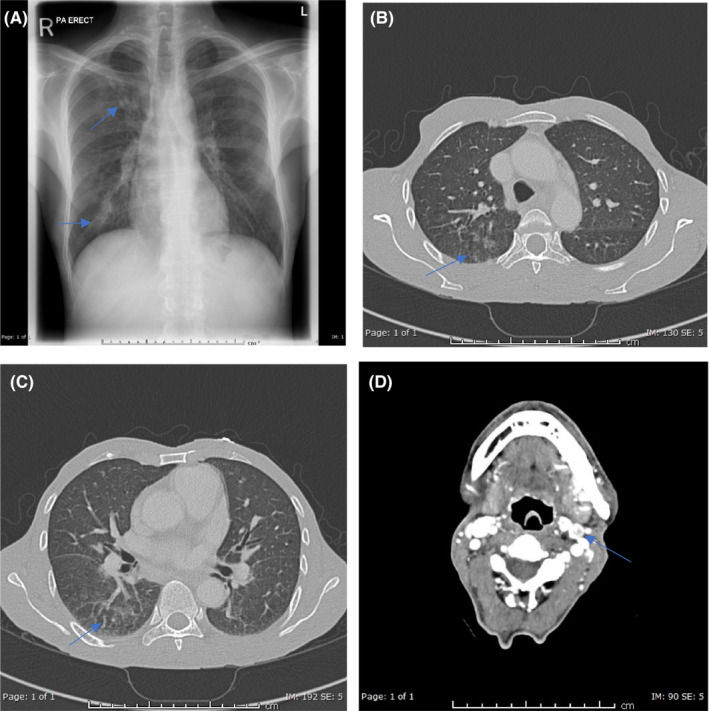
A, Chest X‐ray (PA view) showing small patchy areas of airspace shadowing in the right lung base and in the right infraclavicular region to a lesser extent (arrows). B, C, Axial CT of the chest lung window showing small patchy areas of ground‐glass opacities and nodular infiltration in the posterior segment of the right lung upper lobe (arrow in B) and in the apical segment of the right lower lobe (arrow in C). D, Axial postcontrast study of the neck showing bilateral cervical subcentimeter‐enhancing lymph nodes, some of which show central nonenhancing areas likely representing necrosis (arrow)

Chest radiography showed small patchy areas of airspace shadowing in the right lung base, and minimally in the right infraclavicular region (Figure 1A). Computed tomography (CT) of the neck showed bilateral cervical and supraclavicular enhancing lymph nodes, some of which demonstrated central nonenhancing areas, likely areas of necrosis. The largest lymph nodes were seen at level 2A bilaterally, measuring 8 mm in the short axis dimension (Figure 1D). Chest CT showed right lung ground‐glass nodular infiltration at the posterior segment of the upper lobe and apical segment of the lower lobe, with postero‐basal collapsed consolidation. The left lung showed few nodular opacities (6 × 4 mm) at the lung apex with postero‐basal atelectatic changes. Mediastinal lymph nodes measured the largest at the preaortic space (15 × 11.5 mm) (Figure 1B and C). The Infectious Disease Department (Figure 1D) and the Center of Communicable Disease (CDC) were notified according to our hospital policy, and the patient was started on appropriate treatment including trimethoprim‐sulfamethoxazole and steroids. The patient was discharged after screening for other sexually transmitted diseases. He was to have CD4 count test carried out during follow‐up in the clinic and then to start antiretroviral therapy. Unfortunately, several days later, his blood cultures were positive for *C*. *neoformans*; meanwhile, by then, the patient had returned to his home country. Two weeks later, his sputum and BAL cultures were positive for NTM.

## 3 DISCUSSION

Opportunistic infections caused by bacteria and fungi are common in HIV‐infected patients. Cryptococcosis is a fungal disease caused by *C*. *neoformans* with a high prevalence in immunocompromised patients, especially in those with advanced HIV infection and CD4 T lymphocyte cell (CD4) counts lower than 100 cells/mm^3^, for whom fungaemia has been associated with a poor prognosis.^4^, ^5^ Many other infections have been characterized as opportunistic infections secondary to HIV infection, including but not limited to, infections with *Toxoplasma gondii*, *P*. *jiroveci* pneumonia (PCP), CMV, and *Mycobacterium avium* complex (MAC) infections.

Furthermore, PCP is a major AIDS‐related opportunistic infection, particularly in patients with advanced immunosuppression (CD4 count < 200/µL) in whom HIV infection remained undiagnosed or untreated.^6^

Similarly, NTM are important causes of pulmonary and extrapulmonary diseases in immunosuppressed hosts.^7^, ^8^ To distinguish NTM infections from other opportunistic infections that occurred earlier in the course of HIV infection, such as disseminated MAC infection, NTM infections have been associated with very low CD4 counts, generally below 50 cells/mm^3^.^9^

Simultaneous infections with *C*. *neoformans*, NTM and PCP are rare. They typically occur in immunocompromised individuals, particularly AIDS patients, when the CD4 lymphocyte count is found to be as low as 20/µL, or lower.^10^

In our case, the patient was newly diagnosed with HIV infection. His CD4 count was not known, because he had traveled back to his home country before the test could be requested; however, he was found to have a high viral load (>6 million), which indicates severe disease, and can explain the presence of *C*. *neoformans* fungaemia, which is a rare disease. At the same time, it can explain the co‐infection by *C*. *neoformans* and NTM, which is also infrequent in HIV‐infected patients. In addition, the existence of a third infection with PCP makes our case extremely rare. Furthermore, positive PCR from BAL for both CMV and EBV, and reactive *T*. *pallidum* antibodies, make the presence of other opportunistic and sexually transmitted infections more likely in our patient.

Nowadays, a new test was implemented for the rapid detection of virus, bacterial and fungal infections using microbial cell‐free DNA. This test is designed to create full sequences from the free or fragmented DNA present in the plasma and compares them with stored DNA sequences in the database to find an exact match. Such a technique can scan for the presence of DNA of multiple organisms, including bacterial, fungal, parasitic, and viral in one serum sample at the same time. It has the advantage of rapid results when compared to traditional cultures.^11^, ^12^ Unfortunately, this new generation of tests is not available in our facility.

## 4 CONCLUSION

*Cryptococcus neoformans* fungemia is not common in HIV‐infected patients, and co‐infections with more than three opportunistic pathogens are extremely rare, especially those with *C*. *neoformans*, mycobacteria, and *P*. *jiroveci*. For this reason, in cases of late HIV infection and in advanced stages of immunosuppression, a high level of suspicion of systemic mycoses and concurrent infection by several opportunistic pathogens is required.

## CONFLICT OF INTEREST

None declared.

## AUTHOR CONTRIBUTIONS

EAS and FAR: wrote and edited the manuscript. MSA and HHB: were responsible for the literature review. MA: provided us with labeled radiological images.

## Data Availability

The data that support the findings of this study are available from the corresponding author upon reasonable request.
